# Heme Oxygenase-1 Deficiency Diminishes Methicillin-Resistant *Staphylococcus aureus* Clearance Due to Reduced TLR9 Expression in Pleural Mesothelial Cells

**DOI:** 10.1371/journal.pone.0169245

**Published:** 2017-01-04

**Authors:** Satindra Gahlot, Najmunnisa Nasreen, Judith A. Johnson, Steven A. Sahn, Kamal A. Mohammed

**Affiliations:** 1 North Florida/South Georgia Veterans Health System, Malcom Randall Veterans Affairs Medical Center, Gainesville, Florida, United States of America; 2 Division of Pulmonary and Critical Care Medicine, Department of Medicine, University of Florida, Gainesville, Florida, United States of America; 3 Emerging Pathogens Institute, University of Florida, Gainesville, Florida, United States of America; 4 Division of Pulmonary and Critical Care Medicine, Medical University of South Carolina, Charleston, South Carolina, United States of America; University of Alabama at Birmingham, UNITED STATES

## Abstract

Methicillin Resistant *Staphylococcus aureus* (MRSA) cause pneumonia and empyema thoraces. TLR9 activation provides protection against bacterial infections and Heme oxygenase-1 (HO-1) is known to enhance host innate immunity against bacterial infections. However, it is still unclear whether HO-1 regulates TLR-9 expression in the pleura and modulates the host innate defenses during MRSA empyema. In order to determine if HO-1 regulates host innate immune functions via modulating TLR expression, in MRSA empyema, HO-1^+/+^ and HO-1^-/-^ mouse pleural mesothelial cells (PMCs) were infected with MRSA (1:10, MOI) in the presence or absence of Cobalt Protoporphyrin (CoPP) and Zinc Protoporphyrin (ZnPP) or CORM-2 (a Carbon monoxide donor) and the expression of mTLR9 and mBD14 was assessed by RT-PCR. *In vivo*, HO-1^+/+^ and HO-1^-/-^ mice were inoculated with MRSA (5x10^6^ CFU) intra-pleurally and host bacterial load was measured by CFU, and TLR9 expression in the pleura was determined by histochemical-immunostaining. We noticed MRSA inducing differential expression of TLR9 in HO-1^+/+^ and HO-1 ^-/-^ PMCs. In MRSA infected HO-1^+/+^ PMCs, TLR1, TLR4, and TLR9 expression was several fold higher than MRSA infected HO-1^-/-^ PMCs. Particularly TLR9 expression was very low in MRSA infected HO-1^-/-^ PMCs both *in vivo* and *in vitro*. Bacterial clearance was significantly higher in HO-1^+/+^ PMCs than compared to HO-1^-/-^ PMCs *in vitro*, and blocking TLR9 activation diminished MRSA clearance significantly. In addition, HO-1^-/-^ mice were unable to clear the MRSA bacterial load *in vivo*. MRSA induced TLR9 and mBD14 expression was significantly high in HO-1^+/+^ PMCs and it was dependent on HO-1 activity. Our findings suggest that HO-1 by modulating TLR9 expression in PMCs promotes pleural innate immunity in MRSA empyema.

## Introduction

Pneumonia is a common reason for the infection-related deaths. Methicillin-resistant *Staphylococcus aureus* (MRSA) causes necrotizing pneumonia and empyema thoraces[1]. Pleural mesothelium is a dynamic, metabolically active cellular-monolayer which in addition to maintaining lung barrier functions, also actively contribute in pleural-inflammation, pleural effusion formation, pleural repair, and pleural-defense mechanisms [2,3,4,5,6,7]. PMCs release several CXC and CC chemokines including CXCL2, CXCL8, and CCL2, upon contact to bacterial products or cytokines such as IL-1β or IFN-γ in mice [6,7]. Additionally, PMCs derived chemokines facilitate to the pleural inflammatory cell influx [2,3]. Recently, we have shown that PMCs expressed Toll-like receptors (TLRs) and TLR2 stimulation in PMCs by peptidoglycan (PGN), a TLR2 agonist have been shown to induce β-defensins-2 (BD2) expression suggesting PMC participation in host innate immune response against *S*. *aureus* pathogenesis [3].

Pathogen associated molecular patterns are detected by the host innate immune cells via toll-like receptors (TLRs) among other pattern recognition receptors. TLRs upon ligand binding, transmit signals to intracellular milieu and stimulate important signaling pathways such as mitogen-activated protein kinase pathways and, nuclear factor-κB. TLR signaling contribute to the expression of several inflammatory and antimicrobial genes involved in various pathogenesis including Staphylococcal infections. The CpG-DNA induced TLR9 activation has been demonstrated to provide protection against various infections [8]. The CpG-DNA activation leads to secretion of several cytokines that in turn contribute to non-specific and specific immunity. The use of CpG-DNA as an anti-inflammatory and anti-infectious immunomodulator and as an adjuvant in immunotherapy has been widely-explored [9,10,11]. Empyema pleural fluids contain high bacterial load and bacterial DNA [12,13] thus during empyema PMCs are constantly exposed to bacterial pathogens and their DNA. However, what impact these will have on PMCs in MRSA empyema is still unknown.

The natural antimicrobial peptides released by host cells upon microbial infections are Defensins, Histatins and Cathelicidins. On the basis of the structure and the cells producing them the defensins are parted into α-defensins and β-defensins. The α-defensins are basically produced by neutrophils and Paneth cells in gastro intestinal tract. Histatins are mainly secreted in human saliva [14]. However, β-defensins and Cathelicidins are produced by epithelium. Bacterial pathogens such as *S*. *aureus* are susceptible to human β-defensin-3 (hBD3) [15]. Recently, we have shown the bactericidal activity of mBD2 against *S*. *aureus* [3]. Due to strong antimicrobial activity against *S*. *aureus* the β-defensins are considered as promising candidates, particularly against the systemic infection underlying syndromes such as severe sepsis or empyema.

Heme oxygenase-1 (HO-1), a 32-kDa inducible protein is cytoprotective against oxidative stress. Heme is degraded by the first-rate limiting catalytic activity of HO-1. HO-1 severs the alpha-meso carbon bridge of the b-type Heme molecules via oxidation to yield Biliverdin, carbon monoxide (CO) and free iron[16]. Furthermore, HO-1 also influences a number of cellular processes such as inflammation, and apoptosis, [17]. Defective HO-1 expression due to polymorphism was found to be associated with increased vulnerability to pneumonia in aged population [18]. HO-1 over expressing mice had a survival advantage over HO-1 wild type mice against enterococcus sepsis [19]. In contrast, HO-1 knock-out mice had a significantly decreased survival rate. HO-1 induction rescued mice from Staphylococcus aureus sepsis related lethality [20]. It is believed that the HO-1 mediated protective mechanisms are most likely due to the heme by products the CO and bilirubin-IXα. CO is a key arbitrator of HO-1 function and it embraces anti-inflammatory [21], and anti-apoptotic [22]. In addition CO treatment protected mice against *S*. *aureus* sepsis[23]. Furthermore, CO has been shown to inhibit inflammation in sepsis [24], and also enhancing bacterial clearance by inducing TLR4 expression in macrophages[25]. However, it is still unclear whether and how CO or HO-1 regulates TLR9 expression in PMCs and moderates MRSA empyema. Here we seek to determine the role of HO-1 on TLR9 mediated pleural mesothelial innate immune function in MRSA empyema.

## Materials and Methods

### Reagents and cell culture

TLR9 murine agonist CpG (ODN1826), TLR9 murine antagonist CpG (ODN2088), monoclonal anti-mTLR9 antibody, mouse monoclonal anti-hemagglutinin-tag (HA) Ab, pUNO-mTLR9-HA, pUNO-mcs purchased from Invivogen (San Diego, CA). Avian Myeloblastosis Virus-Reverse Transcriptase (AMV-RT) enzyme, SYBR Green Jump Start ready mix, Lysostaphin, actinomycin-D, and Tricarbonyl-dichloro-ruthenium (II) dimer (CORM2) were obtained from Sigma Aldrich (St. Louis, MO). The Micro-Bicinchoninic Acid assay (BCA) protein assay kit was purchased from Pierce Biotechnology (Rockford, IL). The Cobalt Protoporphyrin-IX (CoPP), Zinc Protoporphyrin-IX (ZnPP), Biliverdin was from Frontier Scientific, Logan, UT. The RNA isolation kit was acquired from Qiagen (Valencia, CA). Murine PMCs were isolated from HO-1^+/+^ and HO-1^-/-^ mice and cultures were established as reported earlier [6].

### MRSA culture

MRSA (USA-3000) was inoculated into Mueller Hinton agar broth complemented with 6 μg/ml of oxacillin and 4% sodium chloride. The cultures were incubated at 35°C for 24 hours and the number of colonies were enumerated by plating on Mueller Hinton agar plates and expressed as CFU/ml. All experimental procedures with MRSA were performed inside the biosafety cabinet with stringent BSL-II precautions and containment.

### Mouse model of MRSA empyema

We established HO-1^-/-^ mice (C57BL/6 back ground) were purchased from Jackson laboratories and required mice were breed in our laboratory. In this study 8 weeks age, (*n* = 6 mice per group) both male and female mice were used. Mice were maintained in micro isolator cages with a Hepa-filter barrier protection in Biosafety level-II housing suite. Animals received sterile food and water *ad libitum*. This study was carried out in accordance with the recommendations in the Guide for the Care and Use of Laboratory Animals of the National Institutes of Health. The animal protocol was approved by the Malcom Randal Veterans Affairs Medical Center Institutional Animal Care and Use Committee (ACORP number: 1155813; project #0015), North Florida/South Georgia Veterans Health System, Gainesville, FL; and all efforts were made to minimize their suffering. Pleural inoculations were performed under anesthesia with 2.5% isoflurane, and the mice were euthanized by exposing them to 2.5% to 5% isoflurane inhalation, followed by cervical dislocation upon confirming lack of toe pinch reflex. HO-1^+/+^ and HO-1^-/-^ mice were intrapleurally infected with 5x10^6^ MRSA CFU in PBS as reported earlier [7]. An additional group of HO-1^+/+^ and HO-1^-/-^ mice were inoculated with equal volume of PBS to serve as controls. The mice were euthanized over time and lungs were harvested for further analysis.

### Treatment of PMCs with HO-1 inhibitors and inducers

HO-1^+/+^, HO-1^-/-^ PMCs were plated in 60mm culture dishes at a concentration of 5x10^5^ cells per dish and maintained at 37°C with 5% CO2 in F-12K complete medium supplemented with streptomycin (100μg/ml), penicillin (100 U/ml), and 10% FBS. PMCs when reached to ∼70% confluence they were transferred to serum-free F-12K medium without antibiotics 16 h before treating them with agonists. The PMC cultures were pre-treated with ZnPP (10μM), CoPP (10μM), CORM2 (100μM) and Biliverdin (20μM) for 1h followed by exposure to MRSA at 10 MOI in serum-free medium (SFM) *in vitro* for indicated time points.

### Quantitative PCR analysis

After treatments, PMC total RNA was isolated using RNeasy Mini Kit, (Qiagen Science, MD, USA). Using oligo-dT primers and enhanced AMV-RT enzyme, 1 μg of total RNA was used to synthesize cDNA by reverse transcription in 20-μl. Real time PCR was performed using 100 ng of cDNA template in triplicate using SYBR Green JumpStart ready mix in a 7500 real-time PCR system (Applied Biosystems, Foster City, CA) as reported earlier[4]. Primers (**Table 1**) to amplify mouse TLRs and mouse Beta-Denfensin-14 (mBD14) were synthesized from Sigma. Mouse Glyceraldehyde-3-Phosphate Dehydrogenase (GAPDH) was amplified as an endogenous control.

**Table 1:** Specific Primers for Real-Time PCR.

Serial No.	Gene	Forward Primer	Reverse Primer
**1.**	**mTLR-1**	GTTGTCACTGATGTCTTCAGC	CTGTACCTTAGAGAATTCTG
**2**	**mTLR-2**	CAGCTTAAAGGGCGGGTCAGAG	TGGAGACGCCAGCTCTGGCTCA
**3**	**mTLR-3**	GAAGCAGGCGTCCTTGGACTT	TGTGCTGAATTCCGAGATCCA
**4**	**mTLR-4**	AGTGGGTCAAGGAACAGAAGCA	CTTTACCAGCTCATTTCTCACC
**5**	**mTLR-6**	AGTGCTGCCAAGTTCCGACA	AGCAAACACCGAGTATAGCG
**6**	**mTLR-9**	CCAGACGCTCTTCGAGAACC	GTTATAGAAGTGGCGGTTGT
**7**	**mBD14**	GTATTCCTCATCTTGTTCTTG G	AAGTACAGCACACCGGCC AC

### Flow cytometry

HO-1^+/+^ and HO-1^-/-^ PMCs were cultured in 60mm dishes, when there were near confluent they were infected with MRSA (1:10, MOI) for 6, 18 and 24 hours along with respective uninfected controls and TLR9 expression was evaluated by flow cytometry as reported earlier[26]. Briefly, PMCs were harvested after indicated time points, rinsed with PBS, and at 4°C they were blocked with 2% BSA in PBS for 20 minutes. PMCs were treated with Fixation and permeablization buffer (BD Biosciences, San Jose, CA, USA) by following the manufacturer’s instructions. Unconjugated rabbit polyclonal IgG1 antibody against TLR9 (Abcam, MA, USA) was used and it was detected with goat anti-rabbit IgG F(ab′)2-FITC secondary antibody. FITC-conjugated goat anti-rabbit IgG1 isotype control (BD Biosciences, San Jose, CA, USA) was taken as negative control. Samples were analyzed using a LSR-II flow cytometer (BD Biosciences, San Jose, CA, USA).

### Determination of MRSA bacterial load

To assess MRSA bacterial load in mice *in vivo* the mice were infected with 5 x10^6^ CFU of MRSA. The lungs, peripheral blood (PB) and pleural fluid (PF) were harvested aseptically at 24h, 48h and 72h post MRSA infection; Lung tissue was minced, viable bacteria (CFU) in the host was determined by plating serial dilutions of the lung tissue homogenate, PB, PF onto Mueller Hinton agar plates in triplicates as reported earlier [7]. Briefly, the test samples were inoculated onto Mueller Hinton agar along with 4% NaCl and 6 μg/ml of Oxacillin. The number of colonies were enumerated and expressed as CFU/ml after incubating for 24–48 hours at 35°C. Similarly, HO-1^+/+^ and HO-1^-/-^ PMCs were exposed to MRSA (MOI 10) in the presence or absence of CoPP and CORM2 or Biliverdin respectively for 24h and CFU were determined as reported earlier[7]. In order to determine if the anti-bacterial activity of PMCs is -TLR9 activation dependent, PMCs were transfected with either TLR-9-HA vector or control-vector and activated with CpG-ODN1826 (TLR9 specific activator), CpG-ODN2088 (TLR9 inhibitor) for 24h in triplicates. The culture supernatants obtained from activated PMCs was incubated with 10^5^ CFU of MRSA in 100μl volume for 3h at 37°C as reported earlier[3]. Parallelly control cultures were maintained without PMC culture media. The cultures were diluted serially, plated on Muller Hinton agar and the MRSA colonies were determined after 24 hours and expressed as percent CFU recovered compared with control MRSA culture.

### Determination of TLR9 expression by western blot analysis

In order to determine the TLR9 expression in PMCs, HO-1^+/+^ PMCs were infected with MRSA in the absence or presence of either CoPP or ZnPP. HO-1^-/-^ PMCs were infected with MRSA in absence and presence of CORM2 or Biliverdin. After 6h of exposure cultures were harvested and TLR9 expression was detected using rabbit polyclonal anti-mouse TLR9 Ab (Abcam, MA, USA) upon SDS-PAGE as reported earlier[3].

### Determination of TLR9 expression by immunohistochemistry (IHC)

TLR9 expression in the pleura and in PMCs cultures was determined by immunohistochemistry as reported earlier[27]. Briefly, lungs were embedded in paraffin; 5μm thick lung sections were prepared. Parallelly, PMCs cultured in chambered slides with and without MRSA infection were also subjected to immunohistochemistry. Endogenous peroxidase activity was blocked by 3% H_2_O_2_ followed by antigen retrieval for 10 min at 95°C (BD Biosciences, San Jose, CA, USA). Sections were blocked with normal goat serum for 30 min at RT. Thereafter, sections were incubated with primary anti-mouse TLR9 antibody (1:200from Abcam, MA, USA) for 45 minutes in humid chamber at 37°C. After washing, diluted biotinylated secondary antibody was applied for 30 minutes followed by conjugation with Streptavidin/Horseradish Peroxidase for another 30 minutes using Avidin-Biotin Complex (ABC) staining kit (Vector Laboratories, Burlingame, CA, USA). Freshly prepared 3’3’-diaminobenzidine (DAB) substrate was applied and monitored for the brown color development. Slides were rinsed, counterstained with hematoxylin for 2 minutes and washed in running water, dehydrated and mounted in VectaMount to observe under microscope.

### TLR9 over expression in PMCs by transfection

In order to delineate the TLR9 mediated signaling, HO-1^+/+^ PMCs were transfected with TLR9 vector as reported earlier[28]. The gene transfer vector, pUNO-mTLR9-HA was used as an expression vector for receptor TLR9 expression, and pUNO-mcs served as a control vector (InvivoGen, San Diego, CA). Transfection was performed with lipofectamine-2000 (Invitrogen, Carlsbad, CA) as per manufacturer’s protocol. TLR9 transfected PMCs were treated with CpG (5μM) for 6, 12 and 24h and RNA was prepared to determine the expression of mouse beta-defensin-14 (mBD14). In another set, transfected cells were pre-treated with CoPP (10μm) or ZnPP (10μM) followed by CpG stimulation for 6h and RNA prepared were subjected to real time PCR for TLR9 and mBD14 expression simultaneously. The over expression of TLR9-HA was also confirmed by Western blotting using anti-HA antibody (InvivoGen, San Diego, CA).

### Statistical analysis

Statistical analyses accomplished using SigmaStat 3.5 (SYSTAT Software, Inc. San Jose, CA). Results were expressed as mean ±SD. Experiments with more than one treatment were assessed using the Kruskal-Wallis and the Mann-Whitney ‘*U’* tests. If the *p*-values were <0.05 the differences were considered statistically significant.

## Results

### MRSA induced significantly higher expression of TLRs in HO-1^+/+^ PMCs

**Fig 1:**
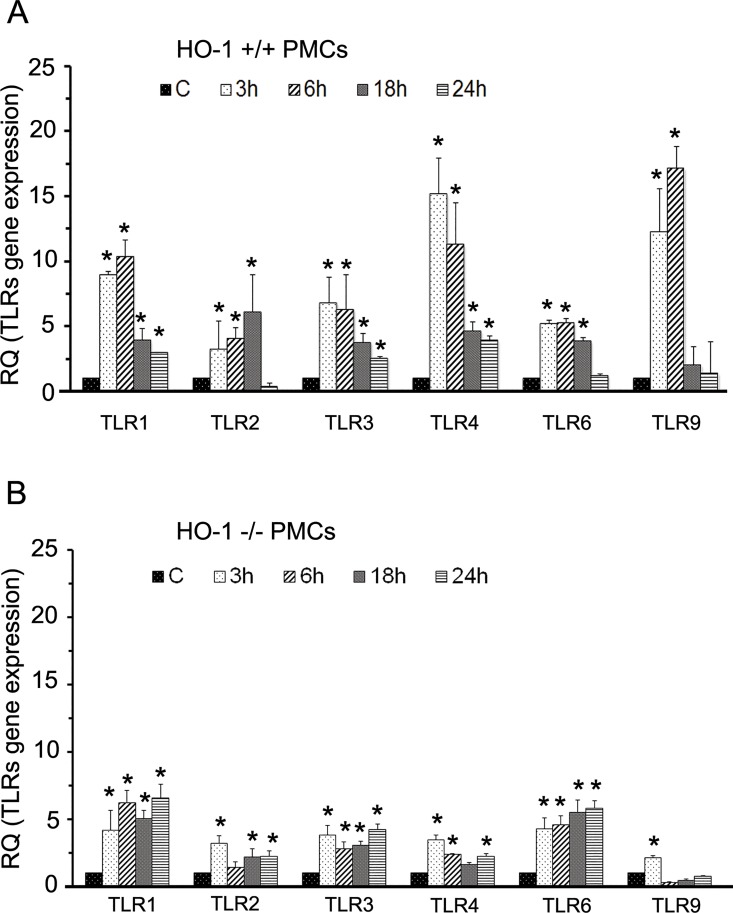
Differential expression of TLRs in HO-1^+/+^ and HO-1^-/-^ PMCs upon MRSA exposure. Panel A and B: TLR expression in HO-1+/+ and HO-1-/- PMCs respectively. HO-1^+/+^ and HO-1^-/-^ PMCs were exposed to MRSA at MOI (1:10) for 3, 6, 18 and 24h in serum-free F-12K medium (SFM). Total RNA was prepared and subjected to reverse transcription and amplification by real-time PCR using primers specific for murine TLR1, TLR2, TLR3, TLR4, TLR6 and TLR9 (refer to Table 1). Data are means ±SD of 3 independent experiments each estimated in triplicate. Statistical significance *p<0.001 compared to un-stimulated (control).

In order to determine the TLR expression in primary cultures of mouse HO-1^+/+^ and HO-1^-/-^ PMCs, cells were infected with MRSA at MOI 10 for 3, 6, 18 and 24 hours. Total RNA was isolated and real- time PCR was performed to determine the TLR specific mRNA expression. The qPCR results showed a significant difference in TLR1, TLR2, TLR3, TLR4, and TLR6, and TLR9 mRNA the expression in MRSA infected both HO-1^+/+^ and HO-1^-/-^ PMCs than compared to resting PMCs (Fig 1). In MRSA infected HO-1^+/+^ PMCs TLR1, TLR4 and TLR9 mRNA expression was several fold higher than MRSA infected HO-1^-/-^ PMCs. Particularly TLR9 expression was very low in MRSA infected HO-1^-/-^ PMCs suggesting HO-1 absence affects in PMC TLR-expression. However, TLR6 mRNA expression in MRSA infected HO-1^-/-^ PMCs was comparable to MRSA infected HO-1^+/+^ PMCs.

**Fig 2:**
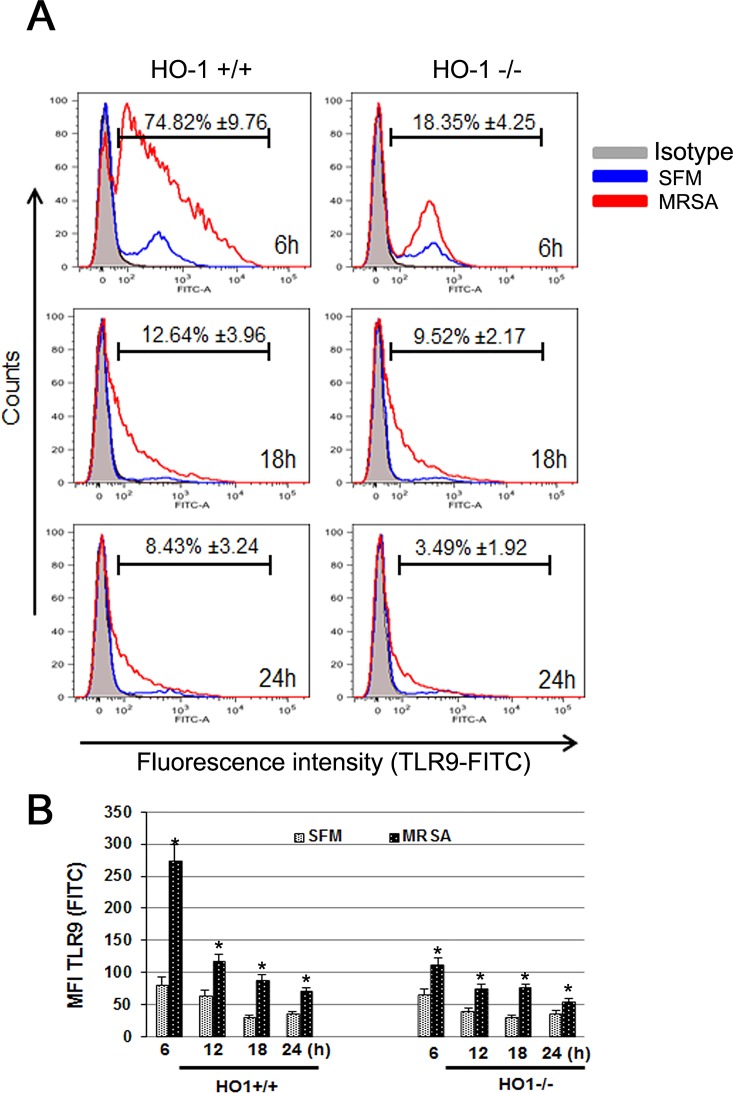
Distinct expression of TLR9 in HO-1^+/+^ PMCs with MRSA infection. Panel A: MRSA induced TLR9 expression in HO-1+/+ and HO-1-/- PMC as measured by Flow Cytometry. HO-1^+/+^ and HO-1^-/-^ PMCs were infected with and without MRSA (MOI of 10) and harvested at indicated times post infection. PMCs were stained for TLR9 as discussed in material and methods section. Overlays are displayed as FITC-TLR9 peak over SFM controls at each time point. FITC–conjugated rabbit secondary IgG antibody was used as isotype control. Bars represent the percent FITC^+^TLR9 cells. Results are representative of three experiments. Panel B: Mean fluorescence intensity of MRSA induced TLR9 expression in HO-1+/+ and HO-1-/- PMCs. Statistical significance *p<0.001 compared to respective serum free media incubated PMC culture (control).

TLR9 expression was also determined by intracellular staining at various time points. Flow cytometric analysis showed a marked increase in TLR9-FITC mean fluorescence value (MFI) in HO-1^+/+^ as compared to HO-1^-/-^ PMCs. The percentages of TLR9-FITC+ PMCs were also determined as compare to their SFM control at each time points respectively. MRSA infection significantly increased TLR-9 expression in PMC when compared to that of control (Fig 2). Highest expression of TLR9 was found at 6h time point; and it was 74.82% ±9.76% in HO-1^+/+^ PMCs whereas in HO-1^-/-^ PMCs only 18.35% ±4.25% were positive. MRSA induced TLR9 expression though declined thereafter in both HO-1^+/+^ and HO-1^-/-^ PMCs, the percent positive cells were significantly higher in HO-1^+/+^ PMCs at all time points tested. The Mean Fluorescence Intensity (MFI) data also revealed significantly higher expression of TLR9 in MRSA infected PMCs than compared to uninfected cells. MRSA induced TLR9 expression was significantly higher in HO-1+/+ PMC than compared to HO-1-/- PMCs and the maximum MFI was noticed at 6h post MRSA infection (Fig 2B).

### MRSA induced TLR9 expression in PMCs regulated by HO-1

**Fig 3:**
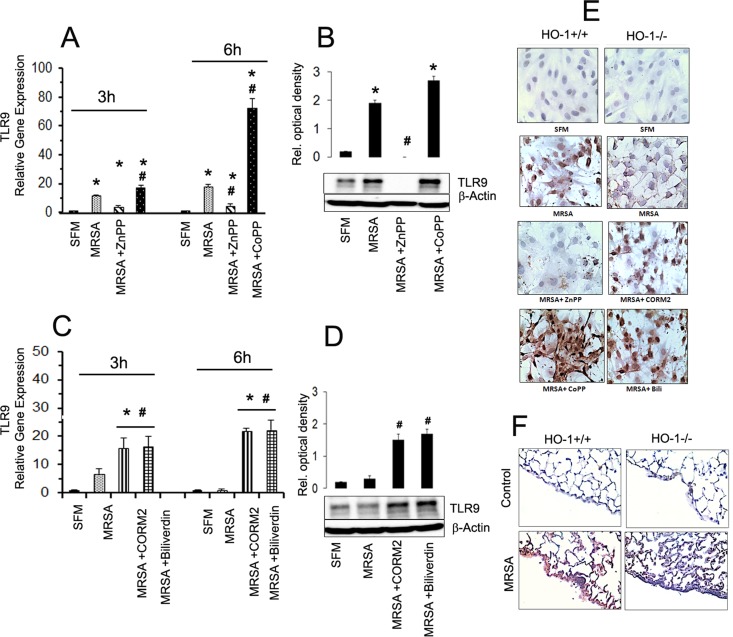
TLR9 expression is HO-1 dependent. Panel A: Wild type PMCs were pre-treated with CoPP (HO-1 inducer) or ZnPP-IX (HO-1 inhibitor) for 1 h followed by MRSA infection for 3 and 6h. Data presented is ±SD of three experiments. Statistical significance *p<0.001 when compared to control; and #p<0.001 when compared to MRSA infected cells. Panel C: Simultaneously, HO-1^-/-^ PMCs were pretreated with CORM-2 (CO donor) or Biliverdin (where indicated) for 1 h followed by MRSA infection for 3 and 6h. Cells were harvested total RNA was isolated and subjected to Real-time PCR for TLR9 mRNA expression. Data presented is ±SD of three experiments. Statistical significance *p<0.001 when compared to control; and #p<0.001 when compared to MRSA infected cells. Panels B and D: Western blot analysis of TLR9 protein expressed in HO-1+/+ and HO-1-/- PMC respectively. β-actin was probed to ensure equal loading of samples. Panel E: TLR9 expression in HO-1^+/+^ and HO-1^-/-^ PMCs as detected immunohistochemistry. HO-1^+/+^ and HO-1^-/-^ PMCs seeded in 4 chamber slides were infected with MRSA (MOI 10) in the presence or absence of either CoPP or ZnPP-IX and CORM2 or Biliverdin for 6 hours as indicated. Extracellular bacteria were lysed with 10μg/ml of Lysostaphin treatment and cells were fixed with 4% paraformaldehyde. Cells were stained using rabbit polyclonal anti-mouse TLR9 antibody as described under material and methods section. Brown color indicates positive staining for TLR9. Pictures presented are the representative image of similar observations from three independent experiments. Panel F: TLR9 expression in pleural mesothelium of control and MRSA empyema mice. Lung tissues from the control and 5x10^6^ MRSA infected HO-1^+/+^ and HO-1^-/-^ mice were subjected to immunohistochemical staining for TLR9 expression as described under material and methods. Brown color indicates positive staining for TLR9. Images are single representative of six similar observations from six mice in each strain.

In order to examine the role of HO-1 on TLR9 expression in PMCs, HO-1^+/+^ and HO-1^-/-^ PMCs were infected with MRSA at MOI 1:10 in the absence or presence of either ZnPP-IX (HO-1 inhibitor) or CoPP (HO-1 inducer). In case of HO-1^-/-^ PMCs, cells were treated with CORM-2 (which releases CO, a HO-1 byproduct after heme metabolism) followed by MRSA infection. Under indicated conditions transcriptional and translational TLR9 expression was evaluated by quantitative RT-PCR and by Western-blot analysis respectively. Our qPCR results clearly demonstrated the drastic down regulation of TLR9 gene expression by ZnPP-IX whereas the CoPP significantly up-regulated the MRSA mediated TLR9 expression in HO-1^+/+^ PMCs (Fig 3A). Western blot analysis data at 6 hours showed increased TLR9 expression in MRSA infected PMCs and ZNPP-IX blocked MRSA induced TLR-9 expression whereas COPP enhanced MRSA mediated TLR9 expression in PMCs (Fig 3B). In HO-1^-/-^ PMCs MRSA infection induced minimal TLR9 expression whereas CORM2 or Biliverdin treatment significantly increased MRSA induced TLR9 gene expression (Fig 3C). In addition, Western blot analysis also showed CORM2 and Biliverdin both up regulated the MRSA mediated TLR9 expression in HO-1^-/-^ PMCs (Fig 3D). These data clearly advocate the significant role of HO-1 in the regulation of TLR9 in PMCs during MRSA infection. IHC staining was also performed to determine the TLR9 expression in both the HO-1^+/+^ and HO-1^-/-^ PMCs. IHC staining displayed highly expressed TLR9 in MRSA infected HO-1+/+ PMCs that were treated with CoPP, however addition of ZnPP reduced the amount of MRSA induced TLR9 expression in HO-1^+/+^ PMCs when compared to HO-1^+/+^ PMCs infected with MRSA (Fig 3E). In contrast, MRSA infection failed to induce TLR9 expression in HO-1^-/-^ PMCs. Moreover, treatment with HO-1 byproducts such as CORM2 and Biliverdin along with MRSA infection increased TLR9 expression in HO-1^-/-^ PMCs. Furthermore, when lungs from both HO-1^+/+^ and HO-1^-/-^ mice were subjected to IHC staining for TLR9 expression, a boisterous expression of TLR9 was noticed in the pleural mesothelium of MRSA infected HO-1^+/+^ mice. Whereas, in HO-1^-/-^ mice infected MRSA, TLR9 expression in pleural mesothelium was scant (Fig 3F). Taken together these results indicate that HO-1 deficiency is connected with poor TLR9 expression in PMCs during MRSA empyema.

### MRSA induces mBD14 expression in PMCs and it is HO-1 dependent

**Fig 4:**
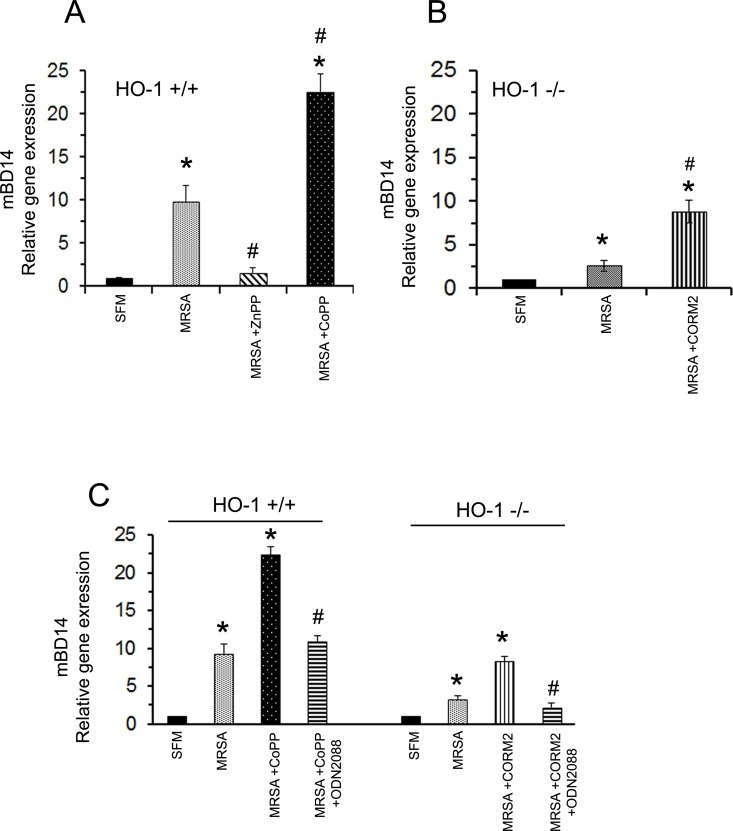
MRSA induced mBD14 expression in PMCs is HO-1 dependent. Panel A: HO-1^+/+^ PMCs were pre-treated with CoPP (HO-1 inducer) or ZnPP (HO-1 inhibitor) for 1 hour followed by MRSA exposure for 6 hours; Data presented is ±SD of three experiments. Statistical significance *p <0.001 significant increases compared to serum free media (SFM) and #p<0.001 significant increase or decrease compared to MRSA infected cells. (Panel B): HO-1^-/-^ PMCs were pretreated with CORM-2 (CO donor) for 1h followed by MRSA exposure for 6h and mBD14 expression in PMCs was determined by Real-time PCR analysis and expressed as the relative gene expression. Data presented is ±SD of three similar experiments. Statistical significance *p <0.001 significant increases compared to serum free media (SFM) and #p<0.001 significant increase compared to MRSA infected cells. Panel C: MRSA infected HO-1^+/+^ and HO-1^-/-^ PMCs were exposed to CoPP and CORM2 respectively and with TLR9 antagonist the ODN2088. PMC mBD14 expression was determined by quantitative RT-PCR analysis. Data presented is ±SD of three experiments. Statistical significance *p<0.001 and compared to serum free media (SFM) #p<0.001 significant decrease compared to MRSA +CoPP treated group in HO-1+/+ PMCs; or MRSA+CORM2 exposed group in HO-1-/- PMCs.

To further investigate the influence of HO-1 with innate immune system we explored mBD14 effector molecule of the innate immune system that offer a potent antimicrobial activity against invading microbes. We noticed MRSA infection inducing mBD14 gene expression in HO-1^+/+^ PMCs that was HO-1 dependent. Inclusion of HO-1 inhibitor ZnPP significantly blocked MRSA induced mBD14 expression, whereas CoPP treatment potentiated MRSA induced mBD14 expression in HO-1^+/+^ PMCs (Fig 4A); CORM2 treatment along with MRSA infection potentiated mBD14 expression in HO-1^-/-^ PMCs (Fig 4B), suggesting HO-1 modulates MRSA induced mBD14 expression in PMCs. Since TLR9 expression was HO-1 dependent, we also sought to examine whether the mBD14 expression noticed was specific to TLR9 signaling. In order to achieve this, we infected the HO-1^+/+^ and HO-1^-/-^ PMCs with MRSA in the presence or absence of CoPP and CORM2 respectively, along with TLR9 antagonist the ODN2088. Interestingly, RT-PCR analysis revealed that addition of TLR9 antagonist significantly reduced the mBD14 gene expression (Fig 4C) suggesting the mBD14 expressed in PMCs is specific to TLR9 activation.

**Fig 5:**
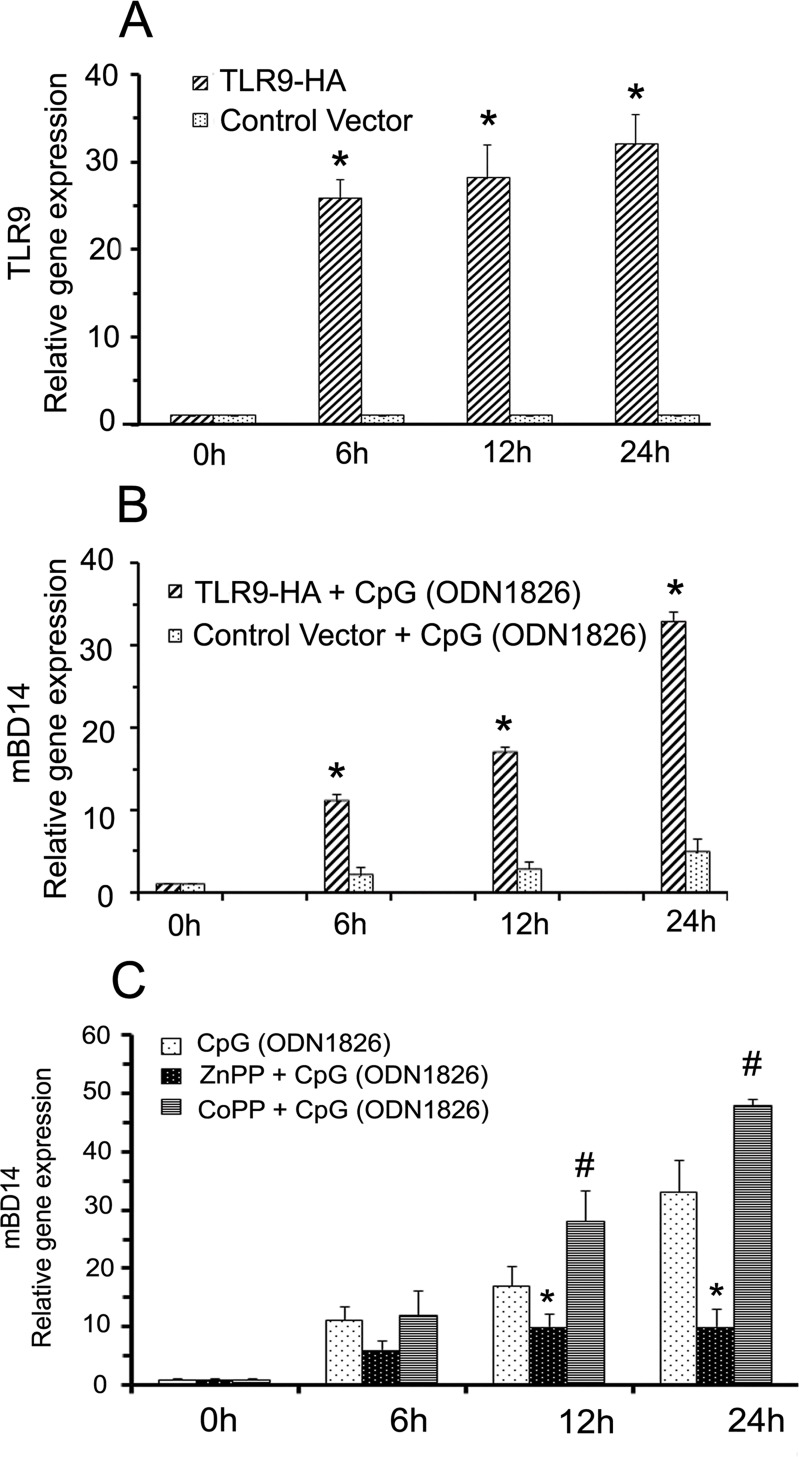
TLR9 receptor mediated mBD14 expression in PMCs is dependent on HO-1. Panel A: RT-PCR analysis of TLR9 expression in pUNO-TLR9-HA vector transfected PMCs. HO-1^+/+^ PMCs were transfected with pUNO-TLR9-HA vector to overexpress the TLR9 receptor or control pUNO-mcs vector as mentioned in materials and methods and the TLR9 expression over time was confirmed by quantitative RT-PCR analysis. Statistical significance *p<0.001 significant increases compared to control vector (pUNO-mcs) transfected PMCs. Panel B: RT-PCR analysis of CpG induced mBD14 expression in TLR9 transfected PMCs. pUNO-TLR9-HA vector and control pUNO-mcs vector transfected PMCs were activated with CpG-ODN1826 over time and mBD14 expression was determined by quantitative RT-PCR analysis. Statistical significance *p<0.001 significant increases compared to control vector (pUNO-mcs) transfected PMCs. Panel C: RT-PCR analysis of CpG induced mBD14 expression in TLR9 transfected PMCs. The pUNO-TLR9-HA vector transfected PMCs were activated with CpG (ODN1826) in presence of either ZnPP or CoPP over time and mBD14 expression was determined by quantitative RT-PCR analysis. Statistical significance *p<0.001 significant decrease/inhibition compared to CpG (ODN1826) treated PMCs and #p<0.001 significant increases compared to CpG (ODN1826) treated PMCs.

In addition, we transfected HO-1^+/+^ PMCs with either mTLR9-HA or control plasmid vector and confirmed TLR-9 gene expression in transfected PMCs by quantitative-PCR. Transfection resulted several fold increases in the TLR-9 expression in PMCs and it was stable for 24 hours (Fig 5A). In order to evaluate if mBD14 expression in PMCs was TLR9 signaling specific, TLR9 transfected and control vector transfected PMCs were stimulation with CpG-DNA and mBD14 expression was determined by quantitative-PCR. These findings reveal a significant increase in the mBD14 expression in CpG-DNA treated TLR9 overexpressed PMCs as compared to CpG-DNA treated control vector transfected PMCs (Fig 5B). Furthermore, we also examined significance of HO-1 in TLR9 mediated mBD14 expression in PMCs. To achieve this TLR9-HA transfected cells were stimulated with CpG-DNA in the absence or presence of either ZnPP or CoPP and mBD14 expression were determined by quantitative-PCR analysis. CpG-DNA induced mBD14 gene expression in PMCs, whereas treatment with ZnPP significantly reduced CpG-DNA induced mBD14 expression; however, treatment with CoPP significantly potentiated CpG-DNA induced mBD14 gene expression in PMCs (Fig 5C). Our data clearly indicated that in PMCs TLR9 signaling mediated mBD14 expression is dependent on HO-1.

### HO-1 deficient mice were vulnerable to MRSA empyema due to poor clearance of bacteria and decreased TLR9 and mBD14 expression

**Fig 6:**
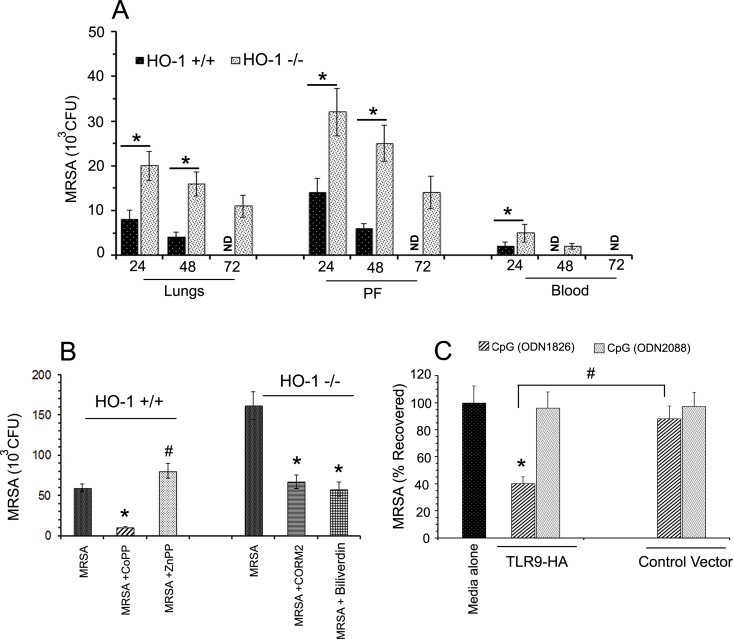
HO-1 deficiency leads to poor bacterial clearance *in vivo* and *in vitro*. Panel A: MRSA CFU levels in HO-1^+/+^ and HO-1-/- mice. HO-1+/+ and HO-1-/- mice infected intrapleurally with 5x10^6^ CFU of MRSA were euthanized at indicated time points post infection. Lungs, pleural fluid (PF) and peripheral blood (PB) harvested was minced, plated on to Muller Hinton agar plates and CFU of MRSA was determined as discussed under material methods. Data presented is Mean ±SD of six mice at each time point in each strain. ND = None detected. Panel B: MRSA CFU levels in HO-1+/+ and HO-1-/- PMCs. HO-1^+/+^ PMCs were infected with MRSA (MOI 1:10) either in presence of CoPP or ZnPP; simultaneously HO-1^-/-^ PMCs were infected with MRSA (MOI 1:10) either in presence of CORM2 or Biliverdin. Cultures were harvested after 24 hours and plated on Muller Hinton agar plates with 1:10, 1:100 and 1:1000 dilutions and CFU of MRSA was determined by counting next day. Data are means ±SD of 3 independent experiments. Statistical significance *p<0.001 a significant decrease compared to MRSA alone exposed cultures; and #p<0.001 a significant increase compared to MRSA +CoPP treated cultures. Panel C: Anti-microbial activity of TLR9-activated/TLR9-inactivated PMC-culture supernatants on MRSA. The pUNO-TLR9-HA vector transfected cells were activated either with CpG (ODN1826) or CpG (ODN2088) for 24h, and culture media mixed with 10^5^ MRSA incubated for 3h. The MRSA colonies were determined after 24h and expressed as MRSA-CFU recovered compared with control media treated cultures. Statistical significance ^$^p<0.001 significant inhibition compared to media alone; *p<0.001 significant increases compared to CpG (ODN1826) activated PMCs; and ^#^p<0.001 significant change between pUNO-TLR9-HA vector transfected and control vector transfected cells.

In order to assess if HO-1 also modulate bactericidal responses *in vivo*, HO-1^-/-^ mice (*n* = 6) and HO-1^+/+^ mice (*n* = 6) were infected with 5x10^6^ MRSA intra-pleurally, and the lungs, pleural fluid (PF), and peripheral blood (PB) were harvested upon euthanasia 24h, 48h and 72h post MRSA infection. Homogenized lung sample, PF, and PB were plated on Muller Hinton agar plates and the number of MRSA colonies enumerated after 24h of incubation. In HO-1^-/-^ mice, we noticed significantly (p<0.001) high bacterial load, in the lungs, and PF, up to 72h post MRSA infection. Whereas in HO-1^+/+^ mice, significantly (p<0.001) low MRSA colonies were noticed in the lungs and pleural fluids up to 48h of infection (Fig 6A). In HO-1+/+ mice MRSA was conspicuously absent in the lung and pleural fluids at 72h post infection. Interesting MRSA was also absent in these mice peripheral blood 48h post infection. However, in HO-1-/- mice MRSA was not recovered from peripheral blood 72h post infection.

To determine the significance of HO-1 on PMCs bactericidal activity *in vitro*, HO-1^+/+^ PMCs were infected with 10 MOI of MRSA in the absence and presence of either CoPP or ZnPP; whereas HO-1^-/-^ PMCs were infected with MRSA with and without CORM2 or Biliverdin. After 24 hours of infection, supernatant containing non-phagocytic or un-ingested bacteria were diluted serially, plated on Muller Hinton agar and the MRSA colonies were determined after 24 hours and expressed as CFU. We noticed significantly less viable MRSA colonies in the HO-1^+/+^ PMC cultures than compared to HO-1^-/-^ PMC cultures. The CFU data from supernatant showed that increased bactericidal activity in CoPP pre-treated HO-1^+/+^ PMCs. Similarly, HO-1^-/-^ PMCs treated with CORM2 and Biliverdin also have higher bactericidal activity than compared to untreated MRSA infected cells (Fig 6B). Similarly, we also determined the anti-bacterial activity of TLR9-activated/inactivated PMCs culture supernatants on MRSA. The TLR9-HA vector, control vector transfected PMCs were activated with either CpG-ODN1826 or CpG-ODN2088 for 24 hours. When the culture supernatants obtained from TLR-9 activated PMCs was incubated with 10^5^ CFU of MRSA, significantly (p<0.001) low MRSA colonies were recovered in CpG-ODN-1826 activated cultures than compared to the culture media obtained from TLR9 specific inhibitor the CpG-ODN2088 treated PMCs (Fig 6C). Interestingly, activation of control-vector transfected PMC with CpG-ODN1826 (a TLR9 specific activator) failed to inhibit MRSA growth and the percent CFU recovered was comparable to media alone treated MRSA cultures. These data clearly suggests that TLR9 inactivation significantly hamper PMC anti-bacterial activity against MRSA.

## Discussion

MRSA causes community associated as well as hospital associated pneumonia and empyema. PMCs are constantly exposed to invading microbial pathogens in empyema. In earlier studies we have reported that during pleural infection PMCs initiate acute inflammatory response by releasing cytokines in the pleural space [2,3,6]. Here we have demonstrated MRSA infection induces TLR9, mBD14 expression in PMCs and it is dependent on HO-1 function. Additionally, the PMCs bactericidal activity during MRSA empyema was dependent on HO-1.

HO-1 function is critical for cytoprotection, anti-inflammation and anti-oxidation. Majority of the HO-1 physiological functions are concomitant to its participation in heme catabolism [29]. Defective HO-1 expression due to polymorphism was found to be associated with increased susceptibility to pneumonia [18]. HO-1 deficiency was also associated severe endothelial injury due to oxidative stress, and increased inflammation [30]. Even though the importance of HO-1 in inflammatory diseases was well defined, HO-1 function during MRSA infection in PMCs remains largely unknown. Thus, in current study we determined the importance of HO-1 on TLR9 signaling and mBD14 response in PMC during MRSA infection. We observed differential expression of TLRs in MRSA infected HO-1^+/+^ and HO-1^-/-^ PMCs. HO-1 deficiency diminished the TLR expression, particularly TLR9 indicating that HO-1 regulates TLRs expression in PMCs thus may contribute to pleural innate immune functions during MRSA empyema. HO-1 pharmacological agents significantly altered MRSA induced TLR9 expression in PMCs suggesting HO-1 may have a distinctive role in TLR9 regulation in the pleura. We also noticed treatment with HO-1 byproducts, such as CORM2 and Biliverdin along with MRSA infection significantly up regulated TLR9 expression in HO-1^-/-^ PMCs. In addition, ZnPP an inhibitor of HO-1 enzymatic activity drastically decreased the MRSA induced TLR9 expression in HO-1^+/+^ PMCs. In mice with *S*. *aureus* sepsis, it was also demonstrated that increased heme catabolism by HO-1 decreases oxidative stress and hence minimizes inflammatory tissue damage and rescues host from Staphylococcal sepsis [20,31]. Furthermore, HO-1 over expressing mice had a survival advantage over HO-1 wild type mice against gram-positive bacterial sepsis; in contrast, HO-1 knock-out mice had a significantly decreased survival rate [19]. Our data suggests that during MRSA infection, HO-1 deficiency leads to poor innate immune response in the pleural space by dampening the TLR9 expression in PMCs.

The pattern recognition receptors such as TLRs recognize microbial pathogen associated molecular patterns (PAMPs) and initiate innate immune defenses against invading pathogens. TLRs signaling triggers β-defensins release and participate in host defenses against invading pathogens. The β-defensins released through TLR signaling, in addition to their antimicrobial function, promote immune cell chemotaxis and thus participate in adaptive immunity. The β-defensins are mostly expressed by the epithelial cells in various tissues and could be induced by pro-inflammatory stimuli [32]. In earlier studies we have demonstrated that mouse primary PMCs express TLRs when exposed to staphylococcal peptidoglycans, produce mBD2 in TLR2-dependent manner and contribute to innate immune defenses [3]. Furthermore, in mice TLR2-deficiency promoted susceptibility to *S*. *aureus* sepsis [33]. Recently, the mouse beta-defensin-14 (mBD14) based on function and structural similarities is identified as human β-defensin-3 (hBD3) orthologue [34]. Impaired hBD3 expression was associated with persistent nasal carriage of *S*. *aureus* [35], and persistent *S*. *aureus* colonization in the skin [36]. The mBD14 expression has been detected in various tissues including upper and lower respiratory tract, colon and spleen. TLR ligands (TLR4, TLR9 and TLR3) have been demonstrated to up regulate mBD14 mRNA expression [37]. Moreover, its expression is regulated by intracellular pattern recognition receptor (PRR), nucleotide-binding oligomerization domain-2 (NOD2), and caspase recruitment domain-5 (CARD5). Furthermore, TLR3 and TLR9 ligands were observed to promote mBD14 expression in murine epithelial cell line [37]. The mBD14 exhibits antimicrobial activities against gram-negative as well as gram-positive bacteria. In the present study, we found that MRSA infection induces mBD14 expression in PMCs and it is HO-1 dependent. In a similar study, Hinrichsen, et al 2008, also noticed mBD14 was effective eliminating *S*. *aureus*/MRSA [34]. Furthermore, we also found significantly reduced bactericidal activity against MRSA in HO-1^-/-^ PMCs as compare to HO-1^+/+^ PMCs. Moreover, addition of HO-1 inducers further enhanced the bactericidal activity in HO-1^+/+^ PMCs, indicating HO-1 modulates PMC bacterial clearance during pleural infection.

Empyema pleural fluids contain excess microbial pathogens and bacterial DNA [12,13]. TLR9 acts as a sensor for bacterial DNA which has abundant un-methylated CpG-nucleotides. However, it is not clear if CpG-DNA has any effect on PMCs innate immune functions. Therefore, we investigated if CpG-ODNs activation of TLR9 leads to mBD14 expression in PMCs. We noticed TLR9 activation by CpG-ODN treatment inducing mBD14 expression in PMCs. In addition, introduction of TLR9 antagonist blocked CpG-ODNs induced mBD14 expression in PMCs suggesting that mBD14 released from PMCs was TLR9 specific response. Similarly, we also determined the bactericidal activity of TLR9 dependent PMCs derived mBD14 against MRSA. The TLR9 activated PMCs significantly blocked MRSA growth and blocking TLR9 activation nullified this inhibitory response suggesting that CpG induced TLR9 dependent mBD14 may inhibit MRSA. The TLR9 agonists, (the CpG-ODNs) are known to trigger innate immune responses in several immune cells including Natural Killer cells, Dendritic Cells (DCs), and macrophages; and are also known to promote adaptive immune responses, such as Th1 polarization, cytokine release, and inhibition of interferons [8,14]. Moreover, TLR9 has been exploited as the important receptor facilitating the interferon induction in DCs during *S*. *aureus* infection, elucidating the significance of TLR9 in host-innate immunity to counter *S*. *aureus* [38]. TLR9^-/-^ mice were found to be susceptible to meningococcal sepsis due to high bacteremia [39]. These observations further explain that TLR9 has a major role in microbial infections.

*S*. *aureus* activates innate immune function via multiple mechanisms including the TLRs, TNFR1; and NLRP3 and NOD2 [38,40,41]. The boisterous inflammatory reaction accompanying with severe Staphylococcal pneumonia may be explained by these multiple redundancies of pro-inflammatory gene activation. Several studies have implicated the role of TLRs on phagocytosis and intracellular bacterial survival. Recently, stimulation of TLR2, TLR4 and TLR9 with their respective ligands: In microglial cells Pam3CSK4, LPS and CpG-ODNs augmented the phagocytosis and intracellular killing of *Chlamydia* [42]. Furthermore, in murine pneumonia TLR9-mediated DCs and macrophage responses effectively cleared the bacteria from the lungs [43,44]. Nevertheless, HO-1–derived CO has been demonstrated promoting bacterial elimination [29]. We noticed CoPP treatment significantly increasing mBD14 expression in MRSA infected HO-1^+/+^ PMCs and CORM2 treatment significantly increasing mBD14 expression in MRSA infected HO-1^-/-^ PMCs suggesting HO-1 regulates mBD14 expression in PMCs and thus modulates host innate immune responses in the pleura.

## Conclusions

Here we have demonstrated the relevance of HO-1 in the PMCs innate immune defenses against MRSA infection. Our experiments elucidated the mechanisms of HO-1 axis in TLR9/mBD14 regulation in PMCs and its protective effect in MRSA empyema. The HO-1 deficient mice displayed reduced bacterial clearance compared to the HO-1^+/+^ mice. Moreover, TLR9-mediated responses may serve as a means to augment antibacterial immunity in MRSA pneumonia or empyema, and these data may facilitate move forward in the development of new treatment strategies against MRSA infection.
